# Characteristics of the *Clostridium difficile* cell envelope and its importance in therapeutics

**DOI:** 10.1111/1751-7915.12372

**Published:** 2016-06-17

**Authors:** Joseph A. Kirk, Oishik Banerji, Robert P. Fagan

**Affiliations:** ^1^ Krebs InstituteDepartment of Molecular Biology and BiotechnologyUniversity of SheffieldSheffieldS10 2TNUK

## Abstract

*Clostridium difficile* infection (CDI) is a challenging threat to human health. Infections occur after disruption of the normal microbiota, most commonly through the use of antibiotics. Current treatment for CDI largely relies on the broad‐spectrum antibiotics vancomycin and metronidazole that further disrupt the microbiota resulting in frequent recurrence, highlighting the need for *C. difficile‐*specific antimicrobials. The cell surface of *C. difficile* represents a promising target for the development of new drugs. *C. difficile* possesses a highly deacetylated peptidoglycan cell wall containing unique secondary cell wall polymers. Bound to the cell wall is an essential S‐layer, formed of SlpA and decorated with an additional 28 related proteins. In addition to the S‐layer, many other cell surface proteins have been identified, including several with roles in host colonization. This review aims to summarize our current understanding of these different *C. difficile* cell surface components and their viability as therapeutic targets.

## Introduction

*Clostridium difficile* is a Gram positive, spore‐forming, anaerobic bacterium and is the leading cause of antibiotic‐associated nosocomial diarrhoea (Rupnik *et al*., 2009). Although the use of antibiotics has undoubtedly had an enormous positive impact on human health over the past seven decades, it is unfortunate that their use is also the main risk factor for *C. difficile* infection (CDI). Hospitalized patients are frequently treated with broad‐spectrum antibiotics, both as prophylactics and to treat infection, resulting in catastrophic damage to the gut microbiota (Dethlefsen *et al*., 2008). *C. difficile* can exploit the resulting dysbiosis to colonize and proliferate in the gut (Lawley *et al*., 2009).

*C. difficile* pathogenesis is a three‐step process that begins with disruption of the gut microbiota (Smits *et al*., 2016). This is followed by germination of indigenous or ingested spores, which starts the colonization phase – involving the attachment of the bacterium to the host intestinal epithelium and multiplication both at the surface and in the lumen. Colonization is required for the final phase of virulence, the release of toxins and the onset of disease symptoms. Most *C. difficile* clinical isolates produce two related toxins, TcdA and TcdB, that belong to the large clostridial cytotoxin family (Jank and Aktories, 2008). Both of these toxins act by glucosylating small GTPases including Rho, Rac and Cdc42. The action of these toxins is responsible for the clinical manifestations of disease, ranging from mild diarrhoea through to life‐threatening inflammatory complications such as pseudomembranous colitis and toxic megacolon (Smits *et al*., 2016). Thirty‐day mortality can exceed 30% in elderly populations (McGowan *et al*., 2011). Since 2001, the emergence of highly transmissible epidemic strains and advances in the availability of genetic tools has increased interest and rapidly advanced our understanding of this important pathogen (McDonald *et al*., 2005; Ng *et al*., 2013; Dembek *et al*., 2015). Despite these improvements, our understanding of *C. difficile* virulence is still in its infancy. It is clear that bacterial cell surface components will be crucial in the interaction between the bacterium and the host. However, the molecular details of these interactions are still largely uncharacterized.

Recent studies focussing on the *C. difficile* envelope have identified and characterized several cell wall polymers, as well as numerous surface proteins (Table 1). Many of these macromolecules are unique to *C. difficile* making the cell envelope a prime target for the development of species‐specific therapeutics. This review will describe our current understanding of *C. difficile* cell envelope architecture, highlighting the potential for novel drugs and vaccines to treat and prevent CDI.

**Table 1:** Therapeutic potential of cell envelope components

Target	Immunogenic	Vaccine formulation	*In vitro* characterization of mutants	*In vivo* characterization of mutants	*Clostridium difficile*‐specific	Other comments	Source
PS‐I	Y	Toxin B conjugate vaccine (untested)	N	N	Y	Only present in a minority of *C. difficile* strains	Ganeshapillai *et al*. (2008), Jiao *et al*. (2013)
PS‐II	Y	CRM_197_ and toxin fragment conjugate vaccines	N	N	Y	Found in all tested *C. difficile* strains. Anchors the essential protein SlpA to the cell surface	Oberli *et al*. (2011), Adamo *et al*. (2012), Romano *et al*. (2014)
PS‐III	Y	HAS and ExoA conjugate vaccines	N	N	PS‐III antibodies cross‐react with other members of the Clostridia		Cox *et al*. (2013), Martin *et al*. (2013)
SlpA	Y	Various vaccination routes with various adjuvants	N	N	Y	Most abundant protein of the S‐layer. This protein is essential and may act as an important colonization factor. SlpA is therefore an interesting target for antimicrobial therapies, although vaccine studies have yielded inconclusive results	Calabi *et al*. (2002), O'Brien *et al*. (2005), Ni Eidhin *et al*. (2008), Ryan *et al*. (2011)
Cwp84	Y	Subcutaneous, rectal and intragastric routes of vaccination with various adjuvants. Oral vaccine also tested	Mutants display poor growth *in vitro*	Mutant remains fully virulent in the hamster model of infection	Y	Although inactivation of Cwp84 does not reduce virulence, vaccination of hamsters with Cwp84 provides protection and results in greater survival rates when compared with control groups	Kirby *et al*. (2009), Pechine *et al*. (2011), Sandolo *et al*. (2011)
CbpA	Untested	N	Mutants display no significant decrease in adhesion to immobilized collagen or human fibroblasts	Mutants show no colonization fitness difference in a competitive mouse model	N	CbpA does not appear to be important for *C. difficile* virulence and is not *C. difficile*‐specific. For these reasons CbpA is unlikely to be a valid antimicrobial target	Janoir *et al*. (2013), Tulli *et al*. (2013)
FbpA	Y	N	Mutant displays no difference in adherence to Caco‐2 or HT29‐MTX cell lines	Mutant showed decreased caecal colonization in a monoxenic mouse model and was outcompeted in a dixenic mouse model	N	Fibronectin‐binding proteins are not *C. difficile*‐specific and the role of FbpA in intestinal adherence appears to be minor. Taken together this suggests that FbpA may not be a suitable target for anti‐*C. difficile* therapies	Pechine *et al*. (2005), Barketi‐Klai *et al*. (2011)
PPEP‐1	Untested	N	PPEP‐1 mutants retain CD2831 on their cell surface, increasing binding to a collagen matrix	PPEP‐1 mutants display slightly lower virulence	Y	Due to the reduction in virulence in a PPEP‐1 mutant, PPEP‐1 remains a viable antimicrobial target	Hensbergen *et al*. (2015)
GroEL	Y	Intranasal immunization with recombinant GroEL	Co‐incubation of *C. difficile* with purified protein or anti‐GroEL antibodies reduce adherence to vero cells	N	N	Although vaccination of mice with GroEL reduces intestinal colonization by *C. difficile*, GroEL is a common bacterial protein and no *in vivo* experiments have been described. For these reasons GroEL does not, at the present, appear to be a good anti‐*C. difficile* target	Hennequin *et al*. (2001a,b), Pechine *et al*. (2013)
CD0873	Untested	N	CD0873 mutants are unable to bind Caco‐2 cells	N	Y	CD0873 remains largely uncharacterized and no *in vivo* studies are available. Therefore, although CD0873 mutants show decreased adherence to Caco‐2 cells more research must be Performed before CD0873 can be characterized as a viable anti‐*C. difficile* target	Kovacs‐Simon *et al*. (2014)

## *C. difficile* cell wall

### Peptidoglycan

**Figure 1:**
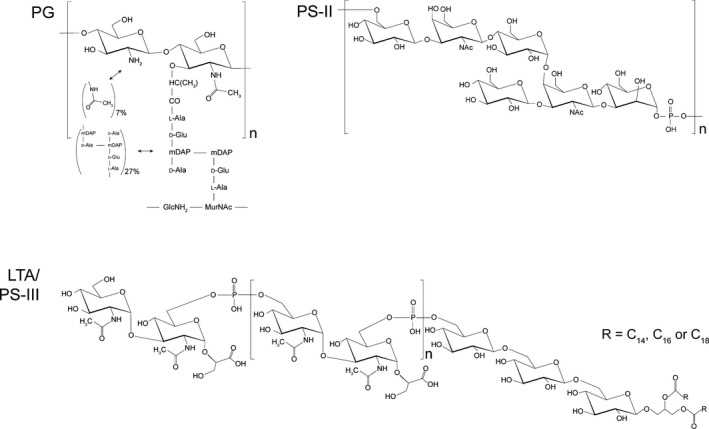
Structure of the conserved cell wall polymers of *Clostridium difficile*. PG:* C. difficile* produces a peptidoglycan characterized by a very high degree of *N*‐acetylglucosamine deacetylation (up to 93%), the stem peptide l‐Ala‐d‐Glu‐A_2_pm‐d‐Ala‐d‐Ala and an unusually high degree (73%) of 3‐3 cross‐links. PS‐II: a conserved cell wall polysaccharide polymer with a core hexasaccharide repeating unit of [→6)‐β‐d‐Glc*p*‐(1→3)‐β‐d‐Gal*p* NAc‐(1→4)‐α‐d‐Glc*p*‐(1→4)‐[β‐d‐Glc*p*‐ (1→3]‐β‐d‐Gal*p* NAc‐(1→3)‐α‐d‐Man*p*‐(1→*P*→]. PS‐III/LTA: a conserved lipid‐anchored cell wall polysaccharide in the extended lipoteichoic acid family with a core repeating unit of [→6)‐α‐d‐Glc*p* NAc‐(1→3)‐[→*P*‐6]‐α‐d‐Glc*p* NAc‐(1→2)‐d‐GroA]. This repeat unit is linked to →6)‐β‐d‐Glc*p*‐(1→6)‐β‐d‐Glc*p*‐(1→6)‐β‐d‐Glc*p*‐(1→1)‐Gro, with the terminal glycerol esterified with C_14_, C_16_, or C_18_ saturated or mono‐unsaturated fatty acids.

Peptidoglycan (PG) is an essential component of the cell wall with pleiotropic functions, including maintenance of cell shape and integrity, and anchoring cell wall proteins (CWP). PG structure is largely conserved, consisting of long glycan polymers cross‐linked by short peptide chains. The polysaccharide backbone is composed of polymers of the β‐1→4 linked disaccharide *N*‐acetylglucosamine‐*N*‐acetylmuramic acid (GlcNAc‐MurNAc). A short peptide stem, which varies between bacterial species, is linked to the D‐lactoyl group of MurNAc [reviewed in (Vollmer *et al*., 2008)]. Muropeptide analysis of digested *C. difficile* PG identified the tetrapeptide stem: l‐Ala‐d‐Glu‐A_2_pm‐d‐Ala (A_2_pm: 2,6‐diaminopimelic acid) (Fig. 1) (Peltier *et al*., 2011). Cross‐linking of the glycan strands in bacteria most commonly occurs via 4‐3 cross‐links catalysed by d,d‐transpeptidases, the essential target of β‐lactam antibiotics (reviewed in Vollmer *et al*., 2008). However, *C. difficile* displays a very high abundance of 3‐3 peptide cross‐links generated by at least two l,d‐transpeptidases. The abundance of 3‐3 cross‐links in *C. difficile* PG is increased by the partial inhibition of d,d‐transpeptidases by ampicillin, suggesting that the l,d‐transpeptidases are insensitive to ampicillin. Despite this, *C. difficile* remains susceptible to ampicillin, suggesting that 4‐3 cross‐linking is essential for PG assembly (Peltier *et al*., 2011).

*N*‐deacetylation or *O*‐acetylation of either GlcNAc or MurNAc introduces further variation in PG structure between bacterial species. *C. difficile* displays very high levels of GlcNAc *N*‐deacetylation (89–93%), while all MurNAc residues remain fully acetylated. The proportion of *N*‐deacetylation observed is higher than that reported for other Gram positive bacteria (Peltier *et al*., 2011). In addition to this naturally high level of PG deacetylation, the percentage of acetylated GlcNAc residues decreases more than twofold in response to the introduction of lysozyme (Ho *et al*., 2014). Lysozyme is a key effector of the innate immune system that cleaves the PG backbone (Bevins and Salzman, 2011); however, PG deacetylation can confer resistance (Vollmer and Tomasz, 2000). Lysozyme induces expression of *csfV*, encoding an extracytoplasmic σ factor, which in turn upregulates expression of the polysaccharide deacetylase PdvA, resulting in further PG deacetylation. *csfV* mutants are severely attenuated in the hamster model of infection (Ho *et al*., 2014).

A notable observation is the presence of a functional *vanG*_*cd*_ cluster in 85% of available *C. difficile* genomes. Vancomycin, an antibiotic of last resort used to treat severe or recurrent *C. difficile* infections, inhibits cell wall synthesis through interaction with the terminal d‐Ala‐d‐Ala of PG precursors (Reynolds, 1989; Johnson *et al*., 2014). Resistance to vancomycin can be conferred via modification of the terminal d‐Ala‐d‐Ala. For example, *Enterococcus faecalis* achieves low‐level resistance through synthesis of d‐Ala‐d‐Ser precursors by enzymes encoded in the *vanG* operon (Depardieu *et al*., 2003). Genes homologous to those in the *vanG* operon have been identified in the majority of *C. difficile* strains tested (Ammam *et al*., 2012; Peltier *et al*., 2013) and are able to synthesize d‐Ala‐d‐ser precursors in response to sublethal concentrations of vancomycin (Ammam *et al*., 2013) although this is debated (Peltier *et al*., 2013). Introduction of the *vanG*_*cd*_ operon into a vancomycin‐sensitive strain of *E. coli* conferred low‐level resistance to vancomycin and a *C. difficile* mutant lacking this cluster displays a slightly lower vancomycin MIC relative to wild‐type (Ammam *et al*., 2013). The *C. difficile* genome also contains genes homologous to *vanW* and *vanZ,* although the function of these genes remains unknown (Ammam *et al*., 2012). Despite this, *C. difficile* remains susceptible to vancomycin treatment. This observation may be due to a number of factors including preferential incorporation of d‐Ala‐d‐Ala precursors by the cell wall synthesis machinery and weak activity of the resistance genes. However, it is possible that genomic alterations allowing *vanG*_*cd*_ *‐*mediated vancomycin resistance could emerge (Ammam *et al*., 2013). Another cell wall synthesis inhibitor, the lipoglycodepsipeptide Ramoplanin, displays efficacy against *C. difficile* in the hamster model and also reduces persistence of spores (Freeman *et al*., 2005). This drug has shown promising results in a phase II trial (Pullman *et al*., 2004) and was fast tracked for development by the US Food and Drug Administration. Ramoplanin was acquired by Nanotherapeutics in 2009 and is due to begin a new phase IIb clinical trial in 2016. An alternative approach to disrupting PG is the use of phage endolysins that degrade the cell wall resulting in cell lysis and death. The ΦCD27 endolysin, CD27L1‐179, can effectively lyse *C. difficile* cells (Mayer *et al*., 2011) by cleaving the bond between MurNAc and the first l‐Ala (Peltier *et al*., 2015). As PG architecture is more highly conserved than proteinaceous phage receptors, the endolysin is effective against diverse strains of *C. difficile*. However, the wider effect of such endolysins on the gut microbiota remains to be tested.

### Secondary cell wall polysaccharides

To date, three anionic polymers have been identified on the cell surface of *C. difficile*. The first described polysaccharide (PS‐I) consists of a branched penta‐glycosylphosphate repeating unit, originally identified in a ribotype 027 strain (Ganeshapillai *et al*., 2008). PS‐I is only found in a minority of strains. The two other polysaccharides, a polymer of hexaglycosylphosphate repeat units (PS‐II) and a lipid bound glycosylphosphate polymer (PS‐III), are more widely distributed and have been found in all strains examined to date (Fig. 1) (Ganeshapillai *et al*., 2008; Reid *et al*., 2012). PS‐I and PS‐II have been described as teichoic acid‐like, although they differ significantly from the simple glycerol phosphate or ribitol phosphate classic teichoic acids (Ganeshapillai *et al*., 2008; Weidenmaier and Peschel, 2008). PS‐III is a member of the extended lipoteichoic acid family (Percy and Grundling, 2014). Although the biological significance of these polymers in *C. difficile* remains poorly understood, PS‐II has been identified as the cell wall ligand that anchors members of the CWP family to the cell surface (see below). Additionally, evidence suggests that secondary cell wall polymer synthesis may be an essential process linked to CWP secretion (Willing *et al*., 2015).

Due to the accessibility of these polymers in the cell wall, they have been investigated as potential vaccine targets. Glycans are T cell‐independent antigens, although when conjugated to a carrier protein, these molecules can elicit a T‐cell memory response (Berti and Adamo, 2013). Anti‐PS‐I antibodies can be detected in sera from healthy horses and chemically synthesized PS‐I has been fused to a subunit of *C. difficile* toxin B to form a potential dual conjugate vaccine (Jiao *et al*., 2013). These preliminary studies demonstrated the potential of cell wall polysaccharides as vaccine candidates. However, as PS‐I is not universal between disparate *C. difficile* strains, PS‐II and PS‐III may represent more promising vaccine targets. Following exposure to *C. difficile*, humans naturally produce anti‐PS‐II antibodies (Oberli *et al*., 2011) and elevated levels of anti‐PS‐II IgM antibodies can be detected in pigs in response to administration of non‐conjugate PS‐II (Bertolo *et al*., 2012). Several conjugate PS‐II vaccines have been developed, using carriers including CRM_197_, a non‐toxic mutant of the diphtheria toxin commonly used as a carrier protein in commercial vaccines (Adamo *et al*., 2012), and *C. difficile* toxin fragments (Romano *et al*., 2014). Both of these conjugate vaccines elicit a PS‐II specific IgG antibody response. Antibodies that react with synthetic PS‐III have also been detected in the blood of *C. difficile*‐infected patients (Martin *et al*., 2013) and conjugate PS‐III vaccines are able to elicit an immune response (Cox *et al*., 2013). However, anti‐PS‐III antibodies also cross‐react with other members of the Clostridium family suggesting that this lipoteichoic acid is not *C. difficile‐*specific.

## S‐layer and cell wall proteins

### *C. difficile* S‐layer

The bacterial surface layer (S‐layer) is a proteinaceous two‐dimensional para‐crystalline array coating the entire cell (Fagan and Fairweather, 2014). S‐layers are usually composed of one or more proteins, called S‐layer proteins (SLPs) that self‐assemble to form the array. SLPs are among the most abundant and metabolically expensive proteins in bacteria that produce them, suggesting that they play a critical role. For example, a typical *C. difficile* cell has a surface area of approximately 18.85 μm^2^ and an S‐layer unit cell of 64 nm^2^ containing two protein subunits (our unpublished data). The S‐layer therefore consists of approximately 590 000 S‐layer subunits, requiring synthesis, export and assembly of 164 subunits per second during exponential growth. *C. difficile* possesses an S‐layer with square‐ordered lattice (Kawata *et al*., 1984), consisting of two distinct proteins, a high‐molecular weight (HMW) SLP (42–50 kDa) and a low‐molecular weight (LMW) SLP (22–38 kDa) (Cerquetti *et al*., 2000). The two SLPs are generated by post‐translational cleavage of a pre‐protein encoded by *slpA* (Calabi *et al*., 2001). The S‐layer appears to be essential, as evidenced by an inability to generate transposon‐mediated insertional mutants within the *slpA* gene (Dembek *et al*., 2015). SlpA has three identifiable subdomains: an N‐terminal secretion signal, followed by the highly variable LMW region and finally the HMW region containing three tandem cell wall binding 2 motifs (CWB2, PF04122) (Fagan *et al*., 2011). The signal peptide directs translocation across the cell membrane via the accessory Sec system (Fagan and Fairweather, 2011), following which the pre‐protein is cleaved by the cell wall localized cysteine protease Cwp84 (Kirby *et al*., 2009; Dang *et al*., 2010), to generate the two SLPs. Following cleavage, the two SLPs form a stable heterodimeric complex (H/L complex; Fagan *et al*., 2009) that self‐assembles to form the mature S‐layer. Extended interaction domains stabilize the HMW and LMW SLP via non‐covalent interactions in the H/L complex (Fagan *et al*., 2009). The CWB2 motifs in the HMW SLP anchor the H/L complex to the cell wall via an interaction with PS‐II (Willing *et al*., 2015). The mechanism by which the H/L complex assembles to form the mature S‐layer is still not understood. It is believed that S‐layer self‐assembly is a thermodynamically driven process (Chung *et al*., 2010) and some SLPs possess a distinct crystallization domain that mediates lateral interactions in the array (Smit *et al*., 2002). However, no such domain has been identified in *C. difficile* SlpA.

A lack of isogenic *slpA* mutants has greatly hampered analysis of *C. difficile* S‐layer function. However, both the LMW and HMW SLPs have been implicated in binding to both cultured Hep‐2 cells and *ex vivo* human gastrointestinal tissue (Calabi *et al*., 2002). Isolated SLPs were also observed to inhibit attachment of *C. difficile* to Caco‐2_BBE_ cells (Merrigan *et al*., 2013). In addition to a possible adhesive role, the S‐layer has also been implicated in host immune activation via TLR4 (Ryan *et al*., 2011). Taken together, these data suggest that the S‐layer plays a crucial role in interactions with the host but detailed analysis of the contribution of the S‐layer to pathogenesis will require isogenic mutants. As the S‐layer is the dominant surface structure on *C. difficile* cells and anti‐SLP antibody has been detected in sera from convalescent patients (Wright *et al*., 2008). Passive immunization with anti‐SLP antibodies also showed some promise in delaying death in the lethal hamster model (O'Brien *et al*., 2005). However, active immunization with purified SLPs alone, or SlpA in combination with cholera toxin, did not result in significant protection using the same model (Ni Eidhin *et al*., 2008; Bruxelle *et al*., 2016). SlpA is highly variable between *C. difficile* strains with at least 12 distinct sequence types known (Dingle *et al*., 2013). An effective therapeutic or vaccine targeting this protein would need to demonstrate protection against all types.

### S‐layer locus

**Figure 2:**
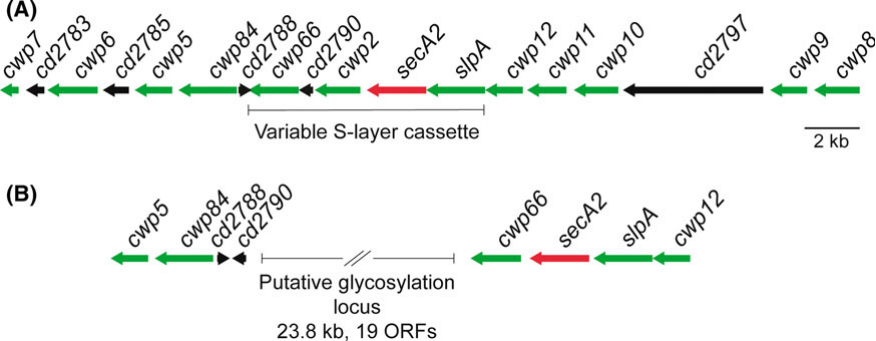
The S‐layer locus. A. *Clostridium difficile* strain 630 encodes 29 cell wall proteins that use the CWB2 (PF04122) motif for non‐covalent anchoring to the cell wall. Twelve of these, including the S‐layer precursor SlpA, are encoded within a single genomic locus (green arrows) that also encodes the S‐layer secretion ATPase SecA2 (red arrow) and five unrelated proteins (black arrows). The core variable S‐layer cassette region is highlighted. An extensive glycan synthesis cluster is located immediately downstream of *cwp7*. It is believed that the proteins encoded in this cluster are responsible for the synthesis of PS‐II (Willing *et al*., 2015). B. One of the 12 identified S‐layer cassettes (cassette type 11) has a 23.8 kb insertion that includes 19 putative ORFs (Dingle *et al*., 2013). Functional predictions of each of the encoded proteins identified all of the activities necessary for the synthesis of a complex glycan and transfer to a substrate. In cassette type 11, the *cwp2* gene is missing and the order of *cwp66* and *cd2790* is reversed.

The *C. difficile* genome encodes 28 paralogues of SlpA (Fagan *et al*., 2011) that make up the CWP family (described below). *slpA* is encoded within a genomic locus including 11 of these paralogues (Fig. 2A) and adjacent to a cluster of polysaccharide synthesis genes thought to be responsible for the synthesis of PS‐II (Willing *et al*., 2015). Genome sequencing of a panel of 57 diverse *C. difficile* strains has identified a 10 kb cassette within the S‐layer locus that displays higher inter‐strain variability than the rest of the locus (Dingle *et al*., 2013). This core variable S‐layer cassette includes the *slpA*,* secA2*,* cwp2* and *cwp66* genes, and 12 distinct cassette sequence types have been identified to date. Interestingly, one of these S‐layer cassettes was found to contain a 24 kb polysaccharide synthesis gene cluster inserted in place of *cwp2* (Fig. 2B). As the *C. difficile* S‐layer is generally not thought to be glycosylated (Qazi *et al*., 2009), it will be very interesting to determine if this SlpA type is glycosylated. The sequence diversity observed between cassette types suggests a strong selective pressure having shaped the antigenic types. This selective pressure can perhaps be attributed to both the host immune response and bacteriophage predation.

### Cell wall protein family

**Figure 3:**
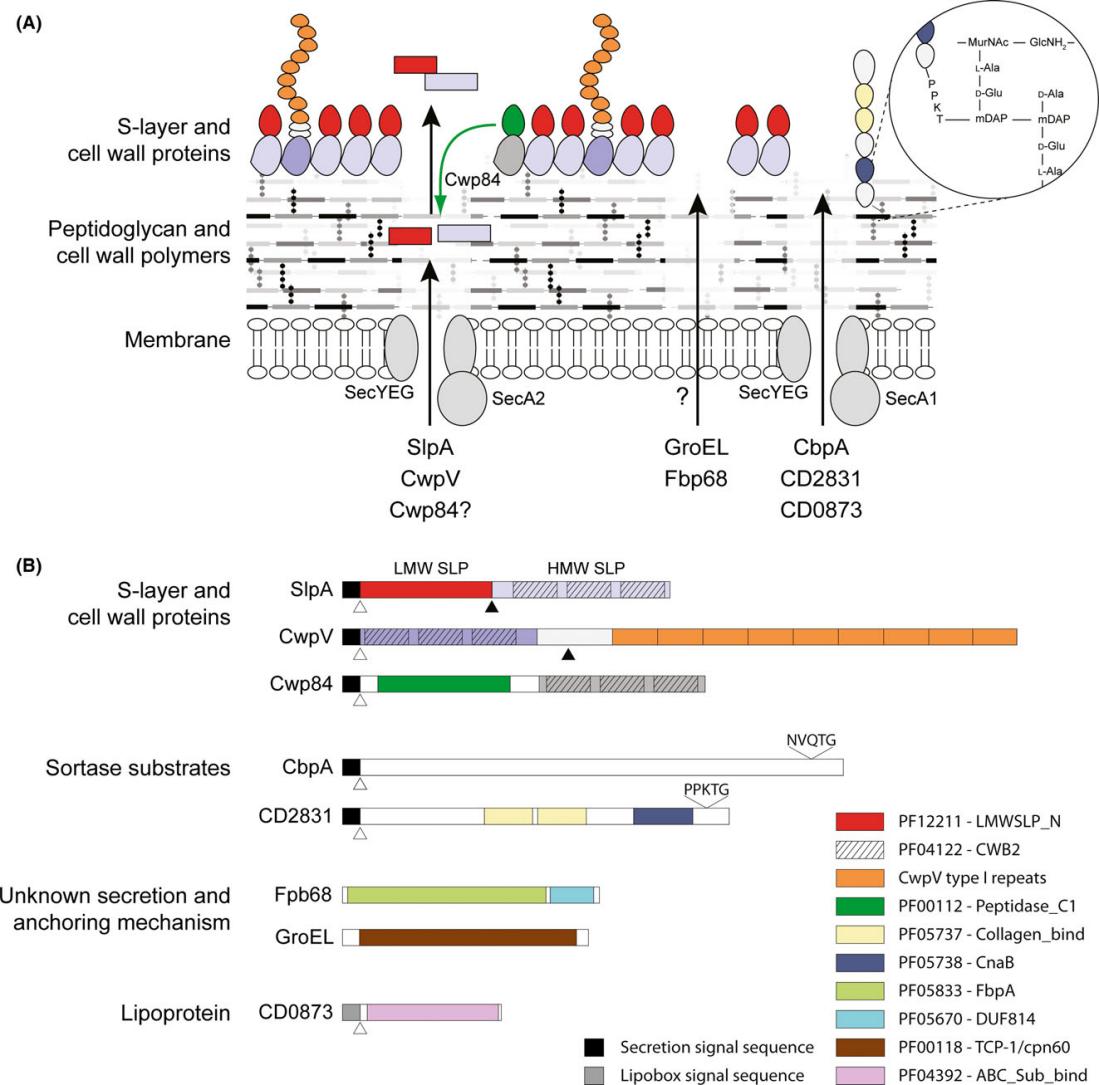
Organization of the *Clostridium difficile* cell envelope. A. *C. difficile* has a normal Gram positive cell envelope with a surface exposed proteinaceous S‐layer on the outer surface. The S‐layer is decorated and functionalized by members of the CWP family; shown are the putative adhesin CwpV and cysteine protease Cwp84. Secretion of the S‐layer precursor SlpA and CwpV are dependent on the accessory ATPase SecA2. Following secretion, SlpA is cleaved by Cwp84 (green arrow), generating the LMW and HMW SLPs. These SLPs form a high‐affinity heterodimer that represents the basic subunit of the S‐layer. CwpV also undergoes post‐secretion processing via an enzyme‐independent auto‐proteolytic mechanism. In addition to the S‐layer and associated CWPs, *C. difficile* possesses numerous other cell surface proteins. The mechanism of secretion and cell wall anchoring of GroEL and Fbp68 (FbpA) is unclear but both can be detected on the cell surface. The lipoprotein CD0873 and sortase‐anchored proteins CbpA and CD2831 are likely secreted via the canonical Sec pathway. Following secretion, CD0873 is attached to the cell membrane via its lipid anchor and the sortase substrates are covalently linked to the peptidoglycan (Thr‐mDap) by the sortase enzyme CD2718. B. Domain organization of the proteins shown in A. N‐terminal secretion signals are shown as black boxes, the CD0873 lipobox is shown in grey and the (lipoprotein) signal peptidase cleavage sites are indicated with white arrows. Post‐secretion cleavage sites are indicated with black arrows. Functional domains demonstrated experimentally or identified using the Pfam database (Finn *et al*., 2016) are also highlighted. The sequence and location of sorting motifs are shown above CbpA and CD2831.

The 28 members of the *C. difficile* CWP family all contain three tandem copies of the CWB2‐anchored surface proteins. Similar families of CWB2‐containing proteins have also been identified in *C. botulinum* and *C. tetani* (Bruggemann *et al*., 2003; Sebaihia *et al*., 2007). *Bacillus anthracis* also has an S‐layer and related family of CWP that share a common anchoring mechanism, the S‐layer homology (SLH) motif (Kern and Schneewind, 2008). The SLH motif is distinct to the CWB2 motif found in *C. difficile* surface proteins but is also found in three tandem copies. The *B. anthracis* SLH motifs adopt a pseudo‐trimeric arrangement forming a three‐pronged spindle that is required for non‐covalent binding to a pyruvylated secondary cell wall polysaccharide (Mesnage *et al*., 2000; Kern *et al*., 2011). Given the similarities to the arrangement of CWB2 motifs within SlpA and the CWPs, it is tempting to speculate that a similar mechanism may be responsible for anchoring these proteins to the anionic polymer PS‐II in the *C. difficile* cell wall (Willing *et al*., 2015). In addition to the CWB2‐anchoring domain, many of the CWPs include an additional domain that is believed to functionalize the S‐layer (Fagan and Fairweather, 2014) (Fig. 3). Only a small number of these CWPs have been characterized in any detail but several have been shown to play crucial roles in the interaction between *C. difficile* and the host.

Cwp66 is a 66 kDa protein with N‐terminal CWB2 motifs. The C‐terminal domain contains an apparently surface‐exposed adhesin that can mediate adherence to Vero cells (Calabi *et al*., 2001; Waligora *et al*., 2001). *cwp66* is transcribed only in early exponential phase as a polycistronic transcript (Savariau‐Lacomme *et al*., 2003).

Cwp84 has a papain class cysteine protease domain (Savariau‐Lacomme *et al*., 2003). Proteolytic enzymes are frequently involved in bacterial colonization process, serving to degrade host proteins including immunoglobulin, nutrient acquisition and processing bacterial proteins necessary in pathogenesis (Maeda, 1996). Purified Cwp84 exhibits proteolytic activity against fibronectin, laminin and type IV collagen, suggestive of a possible role in infection (Janoir *et al*., 2004, 2007). However, the relevance of these host targets is unclear and the activity observed may be opportunistic in nature rather than reflecting underlying biological function. Pull‐down experiments using an inhibitor of SlpA cleavage identified Cwp84 as the SlpA processing protease (Dang *et al*., 2010) and when a *cwp84* insertional knockout strain was constructed, SlpA processing was completely abolished (Kirby *et al*., 2009). The *cwp84* gene is also located close to *slpA* in the S‐layer cassette (Fig. 2A). Taken together, this suggests that SlpA is the principal target of Cwp84. Although potent inhibitors of Cwp84 have been developed (Dang *et al*., 2010; Tam Dang *et al*., 2012), a *cwp84* mutant was fully virulent in the hamster model of acute infection, suggesting that this protease is not a viable antimicrobial target (Kirby *et al*., 2009). As Cwp84 is highly conserved between *C. difficile* strains, it has also been investigated as a possible vaccine candidate (Pechine *et al*., 2011; Sandolo *et al*., 2011). Immunization with Cwp84 induced a specific antibody response and increased survival in the lethal hamster model. However, complete protection was not achieved and further investigation will be required to determine if Cwp84 has potential as a component of an anti‐*C. difficile* vaccine.

A second cysteine protease, Cwp13 is a paralogue of Cwp84, displaying 63% amino acid identity (de la Riva *et al*., 2011). In the absence of Cwp84, Cwp13 can partially substitute in SlpA processing, however, it also displays proteolytic activity against a sequence in the HMW region of SlpA, distinct from that recognized by Cwp84 (de la Riva *et al*., 2011). It has been suggested that Cwp13 plays a role in the turnover of misfolded proteins on the cell surface (de la Riva *et al*., 2011).

CwpV is the largest member of the CWP family and is encoded outside the S‐layer cassette. In addition to N‐terminal CWB2 motifs, CwpV possesses a region of unknown function ending in a flexible serine‐glycine‐rich linker and a C‐terminal region containing 4–9 repeats of 79–120 amino acids (Reynolds *et al*., 2011). Expression of CwpV is phase‐variable, with only 5% of cells in a population expressing the protein *in vitro* (Emerson *et al*., 2009). However, when expressed, CwpV accounts for almost 15% of S‐layer associated protein. Expression of CwpV is controlled by a 195 bp invertible switch located immediately upstream of the gene. The switch is flanked by imperfect 21 bp inverted repeats that can be recombined by the site‐specific recombinase RecV, inverting the intervening DNA (Emerson *et al*., 2009; Reynolds *et al*., 2011). In the ‘OFF’ orientation, a stem loop terminator is formed that prevents transcriptional readthrough. In the ‘ON’ orientation, no stem loop is formed and the gene is expressed. CwpV secretion is mediated by SecA2 (Fagan and Fairweather, 2011), following which, it is cleaved into a ~42 kDa N‐terminal fragment and a 90–120 kDa C‐terminal fragment that form a non‐covalent heterodimeric complex on the cell surface. CwpV cleavage is via intra‐molecular, enzyme‐ and cofactor‐independent autoproteolysis (Dembek *et al*., 2012). The C‐terminal‐repeat region varies between strains and five distinct sequence types have been identified to date, types I–V (Reynolds *et al*., 2011). As with SlpA, antigenic variability of CwpV may be a result of host immune or bacteriophage selective pressure. Indeed, it has been observed that CwpV expression confers protection against bacteriophage infection using a novel mechanism that does not affect phage adsorption but rather prevents phage DNA replication (Sekulovic *et al*., 2015), similar to superinfection exclusion systems. CwpV expression also promotes auto‐aggregation of cells in solid and liquid media (Reynolds *et al*., 2011) similar to those reported in mouse models of colonization (Lawley *et al*., 2009). It is tempting to suggest that CwpV may play a role in host colonization, but further studies will be required to test this.

### Sortase‐anchored proteins

In many Gram positive bacteria, surface proteins are covalently attached to the cell wall by the action of sortases. *Staphylococcus aureus* makes extensive use of this anchoring mechanism and the housekeeping sortase, SrtA, has been well characterized (Schneewind *et al*., 1992). SrtA recognizes a C‐terminal tripartite signal sequence containing a highly conserved pentapeptide cell wall sorting motif, LPxTG. Proteins are then anchored to the cell wall via the catalytic action of a conserved cysteine residue of the sortase which cleaves the LPxTG motif between the threonine and glycine residues and, subsequently, covalently attaches the substrate protein to PG precursors (Fig. 3A) (Perry *et al*., 2002). Six sortase families, with different functions within the cell, have been described, all of which recognize different substrate motifs (Spirig *et al*., 2011). Only one functional sortase gene has been identified in *C. difficile*, a second contains an internal stop codon and is considered a pseudogene (Sebaihia *et al*., 2006). *cd2718* encodes a sortase that displays 32% identity to *S. aureus* SrtB (Donahue *et al*., 2014) and displays structural characteristics of class B sortases (Chambers *et al*., 2015). CD2718 acts on a sorting motif closely related to that of *S. aureus* SrtA, differing at only at the first position, (S/P)PxTG (Donahue *et al*., 2014; van Leeuwen *et al*., 2014). Sortases, although not usually essential for growth, are often required for virulence and are therefore considered targets for new anti‐infective compounds (Cascioferro *et al*., 2014). Small molecule protease inhibitors are able to inhibit the action of *C. difficile* sortase which may aid in the development of new CDI‐specific therapeutics (Donahue *et al*., 2014). However, the use of *C. difficile* sortase as a therapeutic target may prove ineffective as inactivation of *cd2718* does not significantly reduce virulence in the hamster model of infection (Chambers *et al*., 2015).

Studies on *C. difficile* sortase substrates are somewhat limited. Although eight putative sortase substrates have been identified in strain 630 (Donahue *et al*., 2014), attachment to the cell wall has only been demonstrated for a few of these (Chambers *et al*., 2015; Peltier *et al*., 2015). Regulation of surface exposed adhesins is key to the switch between motile and sessile forms (Boyd and O'Toole, 2012). The collagen‐binding protein CD2831 and putative adhesin CD3246 both depend on sortase activity for attachment to the cell wall and are released through the activity of the highly specific and unique protease PPEP‐1 (Zmp1/CD2830) (Hensbergen *et al*., 2015). The bacterial second messenger cyclic‐di‐GMP (c‐di‐GMP) has been associated with the sessile to motile switch (Romling *et al*., 2013). c‐di‐GMP negatively regulates PPEP‐1 expression via a type I c‐di‐GMP riboswitch, and induces *cd2831* via a type II riboswitch (Soutourina *et al*., 2013). Thus, low level of c‐di‐GMP reduces expression of CD2831, while also facilitating release of existing protein from the cell surface and perhaps facilitating the transition from sessile to motile forms (Peltier *et al*., 2015). A PPEP‐1 mutant shows significantly reduced virulence in the hamster model of infection, highlighting the importance of adhesin regulation *in vivo* and suggests that PPEP‐1 is a promising antimicrobial target (Hensbergen *et al*., 2015).

Collagen‐binding protein A (CbpA) has been identified as a putative sortase substrate due to the presence of an apparent SrtB sorting motif (NVQTG) (Tulli *et al*., 2013). Although sortase‐mediated anchoring has not been experimentally confirmed, CbpA is surface exposed. CbpA belongs to the MSCRAMM family (Patti *et al*., 1994), which includes proteins that interact with the host extracellular matrix, and displays high affinity for collagens I and V, the most common components of fibrils. Heterologous expression in *Lactococcus lactis* resulted in surface localization and an increased ability to adhere to both immobilized collagen V and human fibroblasts (Tulli *et al*., 2013). Despite these observations, a *cbpA* mutant displays no significant decrease in adherence to either immobilized collagen or human fibroblasts (Tulli *et al*., 2013). A *cbpA* mutant also showed no significant difference, compared with the parental strain, in colonization fitness in a competitive mouse model (Janoir *et al*., 2013), perhaps due to redundancy with CD2831 (Hensbergen *et al*., 2015) and other adhesion factors. CbpA is therefore unlikely to be an effective antimicrobial target.

### Other cell surface proteins

In addition to the CWP family and sortase substrates, a number of other proteins are localized to the cell surface, through interaction with the cytoplasmic membrane or through uncharacterized mechanisms of cell surface association. Several of these proteins have been identified as important colonization factors during *C. difficile* infection, facilitating adherence to human tissue.

Fibronectin‐binding protein (Fbp68/FbpA) is another member of the MSCRAMM family and is surface associated in *C. difficile*, despite lacking obvious mechanisms of cell surface association or a secretion signal (Hennequin *et al*., 2003). Fbp68 is a manganese‐dependent fibronectin‐binding protein, capable of binding to immobilized fibronectin and cultured vero cells (Hennequin *et al*., 2003; Lin *et al*., 2011). An *fbp68* mutant displayed no significant defect in adherence to either Caco‐2 or HT29‐MTX cells but did show a significant decrease in caecal colonization in a monoxenic mouse model and was outcompeted by the parental strain in a dixenic mouse model (Barketi‐Klai *et al*., 2011). Anti‐Fbp68 antibodies have been found in CDI patient sera suggesting that Fbp68 may perhaps be a useful component of a *C. difficile* vaccine (Pechine *et al*., 2005).

Heat shock proteins have been shown to be important for survival in the host for many pathogenic bacteria (Zügel and Kaufmann, 1999), and *C. difficile* adherence to tissue cultures can be increased through varying stresses including heat shock, acidic pH and low iron levels (Eveillard *et al*., 1993; Waligora *et al*., 1999). GroEL is a member of the Hsp60 chaperonin family and its expression is upregulated in response to all of these stresses (Hennequin *et al*., 2001a). Co‐incubation of *C. difficile* with anti‐GroEL antibodies or purified GroEL significantly decreased adherence of *C. difficile* cells to cultured vero cells, suggesting that GroEL acts as an adhesin (Hennequin *et al*., 2001b). GroEL is also associated with the cell surface, although it lacks a signal sequence or obvious mechanism of cell surface association (Hennequin *et al*., 2001a,b). GroEL is an immunogenic protein in cell wall extracts of *C. difficile* and immunization of mice with recombinant protein reduces intestinal colonization by *C. difficile* (Pechine *et al*., 2013).

CD0873 is a lipoprotein with 21% sequence identity to the PsaA protein of *Streptococcus pneumoniae,* a multifunctional lipoprotein component of an ABC‐type transporter involved in adhesion to the host cell (Rajam *et al*., 2008; Kovacs‐Simon *et al*., 2014). Immunofluorescence microscopy revealed that CD0873 is surface exposed, and likely anchored to the membrane through attached acyl moieties. Although no *in vivo* experiments have been reported, a mutant strain incapable of producing CD0873 is unable to bind Caco‐2 cells, suggesting a role in adhesion to enteric cells. CD0873 is widely conserved (Kovacs‐Simon *et al*., 2014) and therefore represents an interesting antimicrobial target and vaccine candidate.

## The cell surface as a target of phage therapy

Bacteriophage and phage‐like particles have great potential as anti‐*C. difficile* therapeutics (Hargreaves and Clokie, 2014). Although no *C. difficile* phage receptors have been identified to date, the S‐layer, CWPs and cell wall polysaccharides are all likely candidates. Indeed, it is possible that the evolution of S‐layer sequence diversity is driven by phage predation, at least in part. The long evolutionary history of *C. difficile*–phage interactions is apparent in the genome, with numerous prophage (Sebaihia *et al*., 2006), an extensive CRISPR system (Hargreaves *et al*., 2014) and an unusual resistance system based on the phase‐variable protein CwpV (Sekulovic *et al*., 2015). Although no strictly lytic phage have been identified to date, a number of studies have demonstrated the utility of phage therapy against *C. difficile* (Ramesh *et al*., 1999; Meader *et al*., 2010, 2013; Nale *et al*., 2015). ΦCD140 has been successfully used to treat CDI in hamsters, with 14 of 18 hamsters surviving a lethal challenge model (Ramesh *et al*., 1999). However, although the phage therapy was successful, it failed to prevent re‐infection when surviving hamsters were re‐challenged 2 weeks later. Two recent studies have also demonstrated the potential of phage therapy *in vitro*. ΦCD27 dramatically reduced the number of viable *C. difficile* and reduced production of the toxins, TcdA and TcdB, in both batch fermentation (Meader *et al*., 2010) and an *in vitro* gut model (Meader *et al*., 2013), with no apparent effect on the microbiota. However, these studies also highlight the therapeutic limitations of lysogenic phage. In one replicate, ΦCD27 failed to prevent *C. difficile* proliferation in the *in vitro* gut model and this was attributed to early lysogeny (Meader *et al*., 2013). It has also been reported that lysogeny with another phage, ΦCD38‐2, can lead to an increase in toxin production by an epidemic ribotype 027 strain (Sekulovic *et al*., 2011). One possible solution to this potential problem is the use of a varied phage cocktail rather than a single phage species. In one recent study, a panel of seven distinct phage species caused significant lysis of *C. difficile* and prevented appearance of resistant, lysogenic clones (Nale *et al*., 2015). This same phage cocktail delayed onset of symptoms by 33 h in the hamster model of acute disease.

Phage tail‐like particles are also an interesting alternative to traditional phage therapy. Some *C. difficile* strains produce R‐type bacteriocins that display antibacterial activity against other strains of *C. difficile* (Gebhart *et al*., 2012). These bacteriocins have similar structure to the tail filaments of *Myoviridae* phages of *C. difficile*, including ΦCD119 and ΦCD2, and presumably kill *C. difficile* by puncturing the cell envelope and dissipating the membrane potential. These naturally occurring bacteriocins have been modified to increase stability and have demonstrated impressive activity *in vivo* in a mouse model of infection. Importantly, killing is highly specific and these bacteriocins do not perturb the gut microbiota (Gebhart *et al*., 2015).

## Conclusion

*Clostridium difficile* is a major cause of morbidity and mortality worldwide and is the leading cause of antibiotic‐associated diarrhoea. Dysbiosis, normally as a result of antibiotic treatment, is a prerequisite for CDI. Although it is encouraging to note the resurgence in research aimed at the discovery and development of new broad‐spectrum antibiotics, it is clear that we must take a more targeted approach to the treatment and prevention of CDI. The structure and function of the cell envelope is critical to our understanding of bacterial pathogenesis and also in the search for novel therapeutic and vaccine candidates. The recent surge in interest in *C. difficile* pathogenesis and the development of a genetic toolbox for the precise manipulation of the Clostridia has greatly improved our understanding of cell envelope architecture and function. Several components of the envelope show promise as potential drug or vaccine targets, including an unusual PG, at least two conserved secondary wall polymers and a large number of conserved surface proteins. Further study of the cell envelope in years to come will hopefully lead to development of new *C. difficile*‐specific treatments.

## Conflict of interest

R.P.F. received a research grant from AvidBiotics Corp. (South San Francisco, USA) related to the development of *C. difficile* therapeutics.
